# Validity and test-retest reliability of the Brazilian version of the Return-to-work self-efficacy questionnaire

**DOI:** 10.11606/S1518-8787.2018052000237

**Published:** 2018-07-17

**Authors:** João Silvestre Silva-Junior, Ester Paiva Souto, Frida Marina Fischer, Rosane Härter Griep

**Affiliations:** I Instituto Nacional do Seguro Social. Gerência Executiva São Paulo Norte. Agência da Previdência Social Santa Marina. São Paulo, SP, Brasil; II Fundação Oswaldo Cruz. Escola Nacional de Saúde Pública Sergio Arouca. Programa de Pós-Graduação de Epidemiologia em Saúde Pública. Rio de Janeiro, RJ, Brasil; III Universidade de São Paulo. Faculdade de Saúde Pública. Departamento de Saúde Ambiental. São Paulo, SP, Brasil; IV Fundação Oswaldo Cruz. Instituto Oswaldo Cruz. Laboratório de Educação em Ambiente e Saúde. Rio de Janeiro, RJ, Brasil

**Keywords:** Mental Disorders, rehabilitation, Return to Work, Employment, Supported, Self efficacy, Surveys and Questionnaires, utilization, Validation Studies, Occupational Health, Transtornos Mentais, reabilitação, Retorno ao Trabalho, Readaptação ao Emprego, Autoeficácia, Inquéritos e Questionários, utilização, Estudos de Validação, Saúde do Trabalhador

## Abstract

**OBJECTIVE:**

To investigate the validity and test-retest reliability of the Brazilian version of the Dutch questionnaire “*Verwachtingen over werken*”.

**METHODS:**

We analyzed data from a longitudinal study conducted in the city of São Paulo, Brazil, from 2014 to 2016. Participants were 411 workers on sick leave for more than 15 days due to mental disorders. A subsample of 126 participants responded the questionnaire a second time, seven to 21 days later. Factorial and concurrent validities and the test-retest reliability were analyzed.

**RESULTS:**

Most participants were female (71.5%), the average age was 36.7 years; 83.1% had attended 12 or more years of formal schooling; the average length of sick leave was 84 days. The average self-efficacy score tended to be below the scale midpoint. The construct had a two-dimensional structure and the concurrent validity confirmed the original construct. For all items, the test-retest reliability adjusted for prevalence ranged from good (0.70) to almost perfect (0.83).

**CONCLUSIONS:**

While the two-dimensional structure diverges from the original, other parameters were adequate. Application of the Return-to-work self-efficacy questionnaire to Brazilian workers might contribute to the planning of return-to-work process. Additional studies are needed to complement the analysis of the use of this instrument in Brazil.

## RESUMO

**OBJETIVO:**

Analisar a validade e a confiabilidade teste-reteste da versão brasileira do questionário holandês “Expectativas sobre o trabalho”.

**MÉTODOS:**

Foram utilizados os dados de um estudo longitudinal conduzido na cidade de São Paulo, SP, entre 2014 e 2016. Participaram 411 trabalhadores afastados do trabalho por mais de 15 dias devido a transtornos mentais. Uma subamostra de 126 participantes respondeu ao questionário pela segunda vez, com intervalo de sete a 21 dias. Foram analisadas a validade fatorial e concorrente, e a confiabilidade teste-reteste.

**RESULTADOS:**

A maioria dos participantes era do sexo feminino (71,5%), com média de idade de 36,7 anos; 83,1% tinham escolaridade igual ou superior a 12 anos de estudo e o tempo médio de afastamento foi de 84 dias. O valor de autoeficácia médio inclinava-se para valores abaixo do ponto médio da escala. A estrutura fatorial apresentou-se como bidimensional e a validade concorrente confirmou o constructo original. A confiabilidade teste-reteste de cada item, ajustada pela prevalência, variou de boa (0,70) a quase perfeita (0,83).

**CONCLUSÕES:**

Embora a estrutura bidimensional fosse diferente da original, outros parâmetros mostraram-se adequados. O uso do questionário “Expectativa sobre o trabalho” entre trabalhadores do Brasil pode auxiliar no planejamento de processos de retorno ao trabalho. Novos estudos devem ser desenvolvidos para complementar a análise do uso da ferramenta no Brasil.

## INTRODUCTION

Mental disorders, especially depression and anxiety, are the main burden of years lived with disability (YLD)^1^. The time to return to work after sick leaves due to these types of disorders is the second longest following cancer only^2^. Conditions listed in ICD-10 Chapter V (mental and behavioral disorders) were the third main reason for sick pay in Brazil from 2008 to 2011, with an average annual incidence of 34.9 per 10,000 insured individuals^3^. Along this period, the average annual cost to the social security system of new benefits granted for work disability was BRL 186 million^4^. Among the almost 2.4 million new benefits granted in 2016, 8.4% were due to mental and behavioral disorders^4^.

Sick leaves related to mental disorders tend to be longer^5^ and are associated with one of the lowest return-to-work rates^6^. After a 1-year follow-up, a study conducted in Denmark found that 12.7% of employees on sick leave due to mental disorders had not yet returned to work^7^. The management of a worker with psychiatric conditions should start as early as possible to improve their prognosis and return-to-work odds^8^.

Investigation of the factors associated with time to return to work might help professionals involved in rehabilitation detect groups at high risk for long sick leaves. Within this context, return-to-work self-efficacy, i.e., the individuals’ belief on their ability to satisfactorily perform some activity or behavior, is a variable that has received considerable attention in recent years^9^ ^,^ ^10^. Studies that employed self-efficacy as parameter found that it was able to predict the time to^11–13^ and success of return-to-work attempts^14^.

Scientifically validated instruments and scales might provide useful parameters to develop specific approaches for workers attempting to return to work. Several research groups employ a Dutch questionnaire that measures return-to-work self-efficacy after sick leaves due to mental disorders^11–14^. This instrument includes adequate psychometric parameters to assess the workers’ perception of their ability to accomplish their tasks upon returning to work^14^.

The initial steps for cross-cultural adaptation of the Dutch instrument to the Brazilian Portuguese language were described in a previous article^15^. Continuing the assessment of the instrument’s adequacy in a different cultural context, the aim of the present study was to investigate the validity and test-retest reliability of the Brazilian version of the “Return-to-work self-efficacy” questionnaire named “*Expectativas sobre o trabalho*” for use in Brazil.

## METHODS

We analyzed data from a longitudinal study that followed up a population of workers for one year after the start of sick leave, from 2014 to 2016, in the city of São Paulo, Brazil.

Two Social Security Agencies were selected for convenience as source for data collection. The inclusion criteria were as follows: having formal employment relationship registered on the workers’ labor and social security card to establish the participants’ employment situation as permanent; having performed medical legal examination as a requisite to request sick pay; and having been diagnosed with a mental or behavioral disorder (ICD-10 Chapter V) as cause for sick leave longer than 15 days. The exclusion criteria were as follows: having two employment relationships, as this situation would not allow separate assessment of the return-to-work self-efficacy at both jobs; and having returned to work before receiving the invitation to participate in the study, to keep the outcome within the follow-up period in all cases.

Eligible sick pay applicants were invited to participate in the study. The participants were requested to respond to questionnaires after the study aims, benefits, and potential risks were explained. The results were subjected to internal and external psychometric analysis. Two databases corresponding to two different data collection periods (2014–2015, and 2016) were merged, resulting in a total of 411 participants.

The participants were requested to respond to a multidimensional questionnaire that includes the following variables:

- Sociodemographic characteristics: sex, date of birth, education level, and marital status;
- Return-to-work self-efficacy: the participants responded to the Brazilian Portuguese version of the questionnaire “*Verwachtingen over werken*”^14^, entitled “*Expectativas sobre o trabalho*”^15^. On the first application, the questionnaire was attached to a notebook that also included other questions. Seven days later, the questionnaire was sent to the participants by e-mail to respond to it anew (retest). Participants failing to provide an e-mail address were not invited to the retest and were considered as lost to follow-up. A total of 108 participants participated in the retest. The questionnaire requests workers with a mental disorder to imagine themselves returning to work the following day and indicate their expectations in this regard (according to their ongoing emotional state and health condition). The questionnaire comprises 11 statements; respondents should indicate their agreement or disagreement on a six-point Likert scale. Each response is scored from one to six points and the total score ranges from 11 to 66 points;
- *Escala de Autoeficácia Geral Percebida* (AEGP): Brazilian Portuguese version^16^ of General Perceived Self-Efficacy Scale (GPSS)^17^. This instrument assesses perceived self-efficacy to evidence the respondents´ ability to overcome daily difficulties and cope with stressful events in life. It comprises 10 statements, which are responded on a 4-point Guttman scale. Each response is scored from one to four; the total score ranges from 10 to 40. This questionnaire was not subjected to retest;
- *Escala de Depressão, Ansiedade e Estresse* (DASS): We used the Brazilian version^18^ of the short form of the Depression, Anxiety, and Stress Scale (DASS)^19^. This instrument comprises 21 statements responded on a 4-point Guttman scale, which investigates depressive, anxious, and stress-related signs or symptoms in the past week. Each response is scored from one to four points, and these points are used to calculate the domain and global scores. This questionnaire was not subjected to retest.

The participants were asked to authorize the access to their social security records to collect information on the diagnosis (according to ICD-10) reported by medical experts as reason for work disability; we considered the date of start of sick leave as the date of start of work disability.

We performed factor validity and correlation analysis. Factor analysis was meant to analyze the dimensional structure of the scale and its construct validity. The database was divided in two random subsamples, which were separately analyzed in two different stages.

Availability of a previous model led us to begin the identification of dimensions by means of Confirmatory Factor Analysis (CFA). In addition, we performed Exploratory Factor Analysis (EFA) with the second sample to find the model with the best fit to the data in case of inadequacy of the goodness-of-fit indicators.

The criteria to determine the number of factors to be extracted were eigenvalue greater than one and adequacy of the factor structure considering items loading and number of items per factor, in addition to the model goodness-of-fit indicators.

We used the results of EFA to reassess the construct structure with a second CFA considering factors loading and error correlations, and analysis of the internal consistency of convergent and discriminant factor validities.

In all the analyses, we applied the weighted least squares with mean and variance adjusted (WLSMV) estimator, which employs polychoric correlation matrixes adequate to categorical or ordinal variables, available in statistical package Mplus version 7.1. In the assessment of the models, we employed three goodness-of-fit indices which were analyzed together to establish the adequacy of the factor structure: incremental fit indexes Comparative Fit Index (CFI) (good fit > 0.90) and Tuckey-Lewis Index (TLI) (good fit > 0.90) and the parsimonious fit index RMSEA. The RMSEA values range from zero to one; lower values indicate better fit of the model as a function of the sample size based on the population covariance matrix. Values equal to or lower than 0.06 indicate acceptable model fit^20^.

Modification indices (MI) were used to investigate potential residual correlations; values above cutoff point 10.0 are indicative of content redundancy between pairs of items.

Convergent validity was investigated by assessing the standardized loading of each indicator. The criterion adopted was minimum value of 0.5, while values 0.7 or higher were considered ideal. Convergent validity was further investigated by means of average variance extracted (AVE) and composite reliability (CR). For AVE, minimum value 0.50 per construct was considered acceptable; for CR, the minimum accepted value was 0.60 and values above 0.70 were considered adequate^21^.

Discriminant validity was rated present when values were below 0.85 and the variance extracted was higher than the variance shared^21^.

To investigate the correlational validity of data with normal distribution, we analyzed the Pearson’s correlation coefficient calculated for the return-to-work self-efficacy score and compared it to two score scales considered in the original validation of the instrument (AEGP and DASS).

We assessed internal consistency by means of Cronbach’s alpha and investigated changes in its value upon removing items from the scale. The test-retest reliability of each item was tested by means of weighted Kappa-squared statistic with the corresponding 95% confidence intervals (95%CI), and prevalence-adjusted bias-adjusted Kappa (PABAK)^22^. The values to interpret the results for reliability were the following: weak (zero to 0.20), mild (0.21 to 0.40), reasonable (0.41 to 0.60), good (0.61 to 0.80), very good (0.81 to 0.92), and excellent (0.93 to 1.00)^22^.

All the analyses were performed with software Stata SE (version 12.0), except PABAK, which was calculated with program WinPepi (version 11.39).

Participation was voluntary; all participants signed an informed consent form. We requested authorization from the *Instituto Nacional do Seguro Social* (Brazilian National Social Security Institute) to conduct the research at its premises. The study was approved in 2014 by the Research Ethics Committee on Human Beings of the Faculdade de Saúde Pública of the Universidade de São Paulo (School of Public Health of University of São Paulo) (CAAE 23492013.5.0000.5421).

## RESULTS

The sample was mainly composed of women (71.5%), people with high education level (83.1% had 12 or more years of formal schooling), and average age of 36.7 years; the average duration of sick leaves was 84 days. A higher proportion of women and individuals with higher educational level participated in the retest compared to the full sample. Notwithstanding this difference, no significant difference was found in average age, length of sick leave, or self-efficacy score between test and retest (Table 1). The initial CFA (CFA1) tested a unidimensional construct structure but evidenced low factor loading for items #2 (I can’t perform my job well because of my emotional state), #6 (I don’t have any energy left for anything else), and #9 (I can’t solve possible problems at the job). The goodness-of-fit indices were satisfactory, except for RMSEA, which value (0.230) was beyond the recommended one (Table 2).

**Table 1:** Profile of participants in the test-retest psychometric analysis of the Brazilian version of “Return-to-work self-efficacy” questionnaire. São Paulo, Brazil, 2014 to 2016.

Variable	Total sample			Retest sample	
	
	n (%)	Mean (SD)	Var.	n (%)	Mean (SD)
Sex	411			108	
Male	117 (28.5)			20 (18.5)	
Female	294 (71.5)			88 (81.5)	
Age (years)	411	36.7 (8.8)	19–64	108	36.5 (8.2)
Educational level (years of formal schooling)	409			108	
< 12	69 (16.9)			3 (2.8)	
≥ 12	340 (83.1)			105 (97.2)	
Length of sick leave (days)	411	84 (92.2)	15–439	108	83 (88.6)
Return-to-work Self-Efficacy Scale	411	30.6 (10.2)	11–66	108	28.3 (12.6)
General Perceived Self-Efficacy Scale	405	23.1 (7.3)	10–40		
Depression Anxiety Stress Scale	401	44.4 (17.4)	0–63		

SD: standard deviation; Var.: variation

**Table 2:** Result of the initial confirmatory factor analysis (CFA1) of the Brazilian version of “Return-to-work self-efficacy” questionnaire items. São Paulo, Brazil, 2014 to 2016.

Items	CFA1	

	(n = 206)	

	F1	Error
1. I’m able to cope well with the difficulties at the job.	0.831	0.309
2. I can’t perform my job well because of my emotional status.	0.364	0.868
3. I can set boundaries to the tasks performed at the job.	0.775	0.399
4. I can accomplish my tasks performed at the job.	0.850	0.278
5. I can manage emotionally difficult situations at the job.	0.814	0.338
6. I don’t have any energy left for anything else.	0.371	0.862
7. I can concentrate enough on my work.	0.883	0.220
8. I can deal with the hectic routine at the workplace.	0.911	0.170
9. I can’t solve possible problems at the job.	0.183	0.966
10. I’m enough motivated to perform my job.	0.774	0.400
11. I can meet the physical demands of my job.	0.818	0.330

Adjustment indexes		

CFI/TLI	0.885/0.857	
Root mean square error of approximation (RMSEA) (95%CI)	0.230 (0.213–0.248)	
Correlation F1-F2	-	

CFI: Comparative Fit Index; TLI: Tucker-Lewis index

The EFA showed that items #2, #6, and #9 constituted a second dimension, which was confirmed on the second CFA (CFA2). The statements converged into a second dimension, with negative formulation of the statements in common. Therefore, the dimensions were named positive statements and negative statements (Table 3).

**Table 3:** Result of factor analysis of the Brazilian version of “Return-to-work self-efficacy” questionnaire items. São Paulo, Brazil, 2014 to 2016. (n = 206)

Items	EFA			CFA2		
	
	F1	F2	Error	F1	F2	Error
1. I’m able to cope well with the difficulties at the job.	0.846	0.186	0.225	0.865		0.252
2. I can’t perform my job well because of my emotional status	0.054	0.608	0.622		0.639	0.592
3. I can set boundaries to the tasks performed at the job.	0.807	0.045	0.341	0.814		0.337
4. I can accomplish my tasks at the job.	0.907	0.127	0.144	0.920		0.153
5. I can manage emotionally difficult situations at the job.	0.855	0.074	0.254	0.863		0.255
6. I don’t have any energy left for anything else.	0.005	0.813	0.339		0.827	0.315
7. I can concentrate enough on my work.	0.917	0.087	0.163	0.912		0.167
8. I can deal with the hectic routine at the workplace.	0.921	0.910	0.156	0.917		0.159
9. I can’t solve possible problems at the job.	0.022	0.834	0.306		0.814	0.337
10. I’m enough motivated to perform my job.	0.856	0.123	0.268	0.824		0.321
11. I can meet the physical demands of my job.	0.831	0.097	0.313	0.798		0.363

Adjustment indexes						

CFI/TLI	0.985/0.976			0.992/0.990		
*Root mean square error of approximation (RMSEA) (95%CI)*	0.114 (0.093–0.135)			0.073 (0.051–0.940)		
Correlation F1-F2	0.077			0.093		
Correlation between item #10 and #11	-			0.340		

EFA: exploratory factor analysis; CFA: confirmatory factor analysis; CFI: Comparative Fit Index; TLI: Tucker-Lewis Index

Analysis of MI revealed only residual correlation involving item pairs #10 and #11, both being positive statements. This parameter was tested in the last stage of CFA2, and model re-specification included this residual correlation in a freely estimated manner. Factor loading remained above 0.8 in most cases, and all goodness-of-fit indices improved, including significant reduction of RMSEA (from 0.082 to 0.073), which indicates improvement of the model.

The positive statement dimension exhibited excellent convergent validity and internal consistency, with AVE 0.86 and CR 0.98. The negative statement dimension also exhibited good estimates with AVE 0.58 and CR 0.80. In addition, discriminant validity between dimensions was evidenced, since the square root values of the AVE values were higher than their correlation.

The global score on AEGP exhibited dismissible positive correlation with the positive (r = 0.204) and negative (r = 0.137) statement dimensions of the return-to-work self-efficacy scale. The value of Pearson’s coefficient for the scores on DASS and positive (-0.111) and negative (-0.172) statement dimensions of the return-to-work self-efficacy scale was dismissively negative.

The value of Cronbach’s alpha for test and retest was 0.72 and 0.92, respectively. Removal of items #2, #6, or #9 resulted in small changes in Cronbach’s alpha, with values increased in both test and retest (Table 4).

**Table 4:** Cronbach’s coefficient for the scores of the Brazilian version of “Return-to-work self-efficacy” following item removal. São Paulo, Brazil, 2014 to 2016.

Item	Cronbach’s alpha test (n = 411)	Cronbach’s alpha after item removal - test	Cronbach’s alpha retest (n = 126)	Cronbach’s alpha after item removal - retest
	0.722		0.920	
1. I’m able to cope well with the difficulties at the job.		0.652		0.908
2. I can’t perform my job well because of my emotional status.		0.743		0.918
3. I can set boundaries to the tasks performed at the job.		0.654		0.912
4. I can accomplish my tasks at the job.		0.651		0.904
5. I can manage emotionally difficult situations at the job.		0.647		0.906
6. I don’t have any energy left for anything else.		0.721		0.925
7. I can concentrate enough on my work.		0.689		0.907
8. I can deal with the hectic routine at the workplace.		0.686		0.911
9. I can’t solve possible problems at the job.		0.741		0.924
10. I’m enough motivated to perform my job.		0.688		0.910
11. I can meet the physical demands of my job.		0.686		0.908

The weighted Kappa-squared value ranged from 0.19 to 0.70 (weak to good). Adjustment for prevalence and bias (PABAK) increased the value for all items, ranging from 0.70 to 0.83 (good to very good). The three items (#2, #6 and #9) not aligned to the unidimensional structure exhibited the lowest values on both CFA (Table 5).

**Table 5:** Weighted Kappa-squared, 95% confidence interval (95%CI) and prevalence-adjusted bias-adjusted Kappa (PABAK) for test-retest of the Brazilian version of “Return-to-work self-efficacy” questionnaire. São Paulo, Brazil, 2014 to 2016. (Ntest = 411, Nretest = 108)

Items	Weighted Kappa-squared	95%CI	PABAK
1. I’m able to cope well with the difficulties at the job.	0.54	0.37–0.72	0.80
2. I can’t perform my job well because of my emotional status.	0.22	0.01–0.43	0.70
3. I can set boundaries to the tasks performed at the job.	0.61	0.47–0.76	0.86
4. I can accomplish my tasks at the job.	0.70	0.58–0.81	0.86
5. I can manage emotionally difficult situations at the job.	0.48	0.32–0.65	0.78
6. I don’t have any energy left for anything else.	0.40	0.22–0.58	0.75
7. I can concentrate enough on my work.	0.52	0.35–0.69	0.79
8. I can deal with the hectic routine at the workplace.	0.45	0.26–0.64	0.75
9. I can’t solve possible problems at the job.	0.19	0.00–0.38	0.64
10. I’m enough motivated to perform my job.	0.63	0.47–0.78	0.82
11. I can meet the physical demands of my job.	0.51	0.35–0.68	0.75

## DISCUSSION

While preserving the original Dutch context, the Brazilian Portuguese version of the return-to-work self-efficacy questionnaire applied following sick leaves due to mental disorders also considered the characteristic of the Brazilian cultural context^15^. In the present article, we described the results of the psychometric analysis to complete the cross-cultural adaptation of this instrument. The factor structure of the Brazilian version did not exhibit the unidimensional structure of the original version and evidenced inadequate goodness-of-fit. However, we found satisfactory parameters of correlational validity for test-retest reliability and internal consistency.

Momsen et al.^23^ observed that translating scales to different languages and for different cultural contexts might be useful for discussion of similar aspects having theoretical relevance and practical application. After all, attempting to improve work environments and the workers’ health via adjustments of macro-policies or improvement actions at the local level is a common goal for professionals in the occupational health field. The Brazilian version is the first cross-cultural adaptation of the Dutch questionnaire developed to investigate return-to-work self-efficacy after sick leaves due to mental disorders^14^. This fact hinders comparisons to other attempts at verifying the applicability of this instrument outside its original context, namely, a European developed country.

Psychometric assessment of the instrument revealed acceptable validity and test-retest reliability, which justifies its use among Brazilian workers with history of sickness absence due to mental disorders.

In regard to the factor of validity of the scale, the 11-statement version did not exhibit the unidimensional convergence described in Lagerveld et al.’s study^14^. The three statements with negative formulation behaved as a dimension different from the one encompassing the eight positive self-efficacy statements on return-to-work self-efficacy. A possible explanation for this two-dimension structure is the participants’ negative perception of the difficulties with which they would have to cope upon returning to work. The final model exhibited convergent and discriminant validity; the result of the goodness-of-fit indices CFI and TLI were optimal, but the results for RMSEA did not confirm the suggested model. Additional psychometric studies involving application of the analyzed instrument are needed to confirm our findings and further the discussion on its use.

Reichenheim and Moraes^24^ observe that differences between populations subjected to analysis using one and the same instrument might cause variation in its psychometric properties. The group of our study exhibited a remarkable numerical difference between the sexes, and the average age and educational level of the participants were higher compared to the groups that participated in the validation study of the original scale in the Netherlands. However, psychometric divergences relative to the validation of the original Dutch version are not indicative of substantial flaws in the process of adaptation^24^.

Validity analysis by means of the correlation coefficient showed that the Brazilian version was able to capture the general perceived self-efficacy and mental state aspects which compose the theoretical framework underlying the construct. Our results confirm the original construct, which evidenced positive correlation between the two self-efficacy scales and negative correlation between self-efficacy and emotional symptoms. Therefore, the instrument is adequate to its theoretical framework.

The value for internal consistency obtained on the first assessment was lower compared to the original validation study. However, the values of Cronbach’s alpha on retest were very similar to the ones reported in the original Dutch study^14^. Analysis following removal of items showed that the negative statements contributed little or nothing to the internal consistency of the scale.

Intra-examiner longitudinal analysis (test-retest) evidenced good to almost perfect reliability following adjustment for prevalence and bias. The results could have been different were the same method employed for data collection on the first assessment used on the retest. On the first occasion, an investigator was available to dispel eventual doubts on how to respond to the instrument.

An assessment of self-efficacy predicts the time to and success in returning to work^11^ ^,^ ^13^ ^,^ ^14^; investigation of the workers’ expectations might help identify situations which pose greater difficulties to their return to work in the short or medium term. The score on the return-to-work self-efficacy questionnaire might serve to orient interventions targeting the working conditions and process of reintegration with favorable impact on the workers’ expectations on their return to work.

Application of the analyzed questionnaire might also help orient public and entrepreneurial policies by bringing information on the possible limitations of workers to return to work. It might also provide information to professionals from social security agencies and occupational health services. Those services should analyze the relationship between workload and disability of workers on sickness absence for the purpose of planning their return to work. We expect that the analyzed instrument will have a positive influence on social reintegration via work and contribute to monitor the efficiency of this process. In addition, the use of this instrument might make the participation of workers in such planning meaningful^23^.

The predictive power of the return-to-work self-efficacy questionnaire for healthy workers who fall ill and for cases of sick leaves because of other types of diseases affecting workers with mental disorders still remains to be investigated. The access to mental health care services is substandard in Brazil, and the rate of persons under treatment in the metropolitan area of São Paulo is low^25^. Facing this scenario, occupational health services might plan an active role in mental health promotion and early detection of disorders to compensate for the flaws in the primary healthcare system.

Scientifically validated tools, such as the return-to-work self-efficacy questionnaire, might serve to monitor the progression of the attempts to return to work. The use of such instruments might also be useful in the discussion on the factors that influence the permanence of workers in the labor market after returning to work.

Within social security professional rehabilitation services, application of objective parameters for assessment, establishment/follow up of goals, and decision making might be grounded on information provided by studies with workers receiving sick pay.

We sought to minimize the occurrence of selection bias by recruiting participants at two different social security agencies. However, both are located in the city of São Paulo and were selected for convenience, which hinders the generalization of the results as a function of the lack of local representativeness of the sample. No difference was found upon comparing eligible subjects who refused to participate and the ones who accepted to participate, which contributes to the internal validity of the study. Differently, the losses between test and retest did pose a problem, because they had a negative impact on the psychometric validation of the questionnaire. We applied statistical techniques to adjust the findings as best as possible.

The criterion adopted to include workers with mental disorders is one of the strengths of the present study, because the participants’ condition was confirmed at three levels – personal physician, occupational/company physician and social security medical examiner. Nevertheless, both diagnosis of psychiatric disorders and the determination of disability were not free from subjective aspects, which might have interfered by allowing the inclusion of false-positives and exclusion of false-negatives.

Standardized data collection performed by a single interviewer sought to minimize bias. However, the different technique used in the retest might have influenced the results.

## FINAL CONSIDERATION

In the present study, we performed the psychometric analysis of the Brazilian Portuguese version of a Dutch questionnaire that assesses return-to-work self-efficacy after sick leave due to mental disorders. Some of the analyzed parameters diverged from the ones of the original version. Additional studies on the dimensional structure of the scale might confirm our findings.

Application of the questionnaire “*Expectativas sobre o trabalho*” to Brazilian workers might contribute to the planning of return to work for workers with disabling mental disorders. As a function of its easy applicability, this instrument is adequate for use at the various types of worker’ care services. Future studies are needed to complement the analysis of the use of this instrument in Brazil.
